# The Past, Current and Future Research in Cerebellar TMS Evoked Responses—A Narrative Review

**DOI:** 10.3390/brainsci14050432

**Published:** 2024-04-26

**Authors:** Po-Yu Fong, John C. Rothwell, Lorenzo Rocchi

**Affiliations:** 1 Department of Clinical and Movement Neurosciences, UCL Queen Square Institute of Neurology, University College London, London WC1N 3BG, UK; j.rothwell@ucl.ac.uk (J.C.R.);; 2 Division of Movement Disorders, Department of Neurology and Neuroscience Research Center, Chang Gung Memorial Hospital, Linkou Branch, Taoyuan 333, Taiwan; 3 Medical School, College of Medicine, Chang Gung University, Taoyuan 333, Taiwan; 4 Department of Medical Sciences and Public Health, University of Cagliari, 09124 Cagliari, Italy

**Keywords:** TMS, EEG, cerebellar TMS, TEP, cerebellothalamocortical tract

## Abstract

Transcranial magnetic stimulation coupled with electroencephalography (TMS-EEG) is a novel technique to investigate cortical physiology in health and disease. The cerebellum has recently gained attention as a possible new hotspot in the field of TMS-EEG, with several reports published recently. However, EEG responses obtained by cerebellar stimulation vary considerably across the literature, possibly due to different experimental methods. Compared to conventional TMS-EEG, which involves stimulation of the cortex, cerebellar TMS-EEG presents some technical difficulties, including strong muscle twitches in the neck area and a loud TMS click when double-cone coils are used, resulting in contamination of responses by electromyographic activity and sensory potentials. Understanding technical difficulties and limitations is essential for the development of cerebellar TMS-EEG research. In this review, we summarize findings of cerebellar TMS-EEG studies, highlighting limitations in experimental design and potential issues that can result in discrepancies between experimental outcomes. Lastly, we propose a possible direction for academic and clinical research with cerebellar TMS-EEG.

## 1. Communication between the Cerebellum and Cerebrum

The cerebellum, often associated primarily with motor control, encompasses a broader range of functions than commonly acknowledged. Beyond its role in coordinating movement, the cerebellum plays a crucial part in regulation of emotion [1,2,3,4], speech [5,6,7], cognitive function [4,8,9,10], motor preparation [11], and motor learning [12,13,14,15]. This multifaceted involvement stems from the cerebellum’s ability to modify information received from the cerebral cortex and return processed signals [16]. The intricate network between the cerebral cortex and the cerebellum includes widespread projections from various cortical areas, encompassing the frontal, parietal, temporal, and occipital lobes [17,18,19]. The communication between these two regions relies primarily on two neural pathways [16,20]. The first, known as the cerebello-thalamo-cortical (CTC) tract, includes the dentato-thalamo-cortical (DTC) tract and projections from Purkinje cells located in the cerebellar cortex to the dentate nucleus [21]. The DTC tract, originating from the dentate nucleus and terminating in contralateral hemispheres via the contralateral ventral thalamus, serves as the primary relay for transmitting signals processed by the cerebellum [9]. Simultaneously, the projections from Purkinje cells modulate the activities of the DTC tract, contributing to the overall coordination of activity within the circuit [22]. The second pathway, the cortico-ponto-cerebellar (CPC) tract, relays cortical efference to the pons and then the contralateral cerebellum [16,23]. Together, these projections establish a close circuit that not only governs motor coordination but also contributes significantly to non-motor functions, highlighting the cerebellum’s integral role in diverse cognitive and motor processes [5,8,9,12,20,24,25]. 

Investigating the connectivity between the cerebellum and the cerebrum involves various methods, with neuroimaging tools and neurophysiological techniques being commonly employed. Functional MRI (fMRI) is a widely used tool, offering high spatial resolution [26] to explore connectivity between cerebellar substructures and specific cortical areas under different experimental conditions [27,28] or during complex tasks like motor learning [29,30]. However, its low temporal resolution [26] limits its ability to capture early neural activities. Other emerging methods for investigating cerebellar–cortical connectivity are magnetoencephalography (MEG) or high-density electroencephalography (EEG); these provide accurate time resolution, at the expense of limited spatial interpretation of neural generators [31,32]. 

Transcranial magnetic stimulation (TMS) has emerged as a useful tool to investigate the CTC pathway. TMS studies on cerebellar physiology often involve measuring changes in motor evoked potentials (MEP) recorded by surface electromyography (sEMG) using the cerebellar–motor cortex inhibition (CBI) protocol [33,34,35]. The CBI protocol entails conditioning MEPs evoked from the motor cortex with a single TMS pulse applied over one cerebellar hemisphere, usually via a double cone (DC) coil, with a 5–7 ms interstimulus interval (ISI) [33,36,37,38]. By combining it with neuromodulation protocols such as repetitive TMS (rTMS), transcranial direct electric stimulation (tDCS), and transcranial alternative electric stimulation (tACS), the CBI protocol can assess synaptic plasticity mechanisms in the cerebellum [39]. CBI can be probed either at rest or during behavioral tasks involving motor learning [40,41,42] or cognitive tests [43]; these applications are useful to shed light on how the cerebellum responds to sudden changes in motor feedback [44], cognitive processing [45], and visual learning [43]. However, one limitation of CBI is that it relies on MEP recording and it only tests connectivity between the cerebellum and the primary motor cortex (M1). Other non-invasive brain stimulation techniques, such as paired associative stimulation [46,47,48] or cerebellar repetitive TMS [49], may also be useful for indirectly probing connectivity between the cerebellum and non-motor cortex. Alternatively, this issue may be overcome by the combination of TMS and neuroimaging tools, which would allow for direct investigation of more widespread connections between the cerebellum and cerebral cortex and associated non-motor functions of the cerebellum.

## 2. Introduction to TMS and EEG Co-Registration

The development of transcranial magnetic stimulation and EEG co-registration (TMS-EEG) has emerged as a powerful tool in electrophysiology and neuroscience research [50,51,52,53]. TMS-EEG involves recording EEG activity evoked by TMS pulses to an area of the cortex or cerebellum. These signals can then be analyzed in the time domain (TMS-evoked potentials (TEP)), and frequency domain (TMS-induced oscillations). These electrophysiological readouts provide extensive insights into the physiology of stimulated or remote cortical areas [54,55]; they also represent useful biomarkers for neurological diseases [56,57] and for the assessment of the effects of drugs [58,59] or non-invasive brain stimulation techniques [60]. Common target areas for TMS-EEG include M1 [61,62,63,64,65,66,67], the dorsolateral prefrontal cortex (DLPFC) [64,68,69,70], and premotor areas [71,72], where consistent and reproducible results have been reported [73].

Despite the many research applications of this technique, the interpretation of TMS-EEG signals can be made difficult by electrical artifacts and cranial muscle activation induced by coil discharge, as well as by auditory and somatosensory input generated by TMS. The first is caused by the TMS click, which activates the auditory system via air- or bone-conducted sound [74,75] and generates an auditory evoked potential (AEPs) mostly consisting in a vertex negativity occurring around 100 ms and a positive wave at 180–200 ms with [76,77]. Notably, AEPs can be minimized or abolished with the use of masking noise [50,78], ear defenders [76,79], and a layer of sponge underneath the coil [50,56,78,80]. Contamination of TMS-EEG responses by somatosensory evoked potentials (SEP) is more controversial, but it is likely that only contraction of craniofacial muscle and activation of free cutaneous nerve endings in the scalp may be able to induce EEG responses, and that these are represented by a N100/P200 complex, reflecting a saliency-related multimodal response, rather than specific activation of the primary somatosensory cortex [76,81]. Additional ways to control for sensory activation in the context of TMS-EEG include comparison with so-called realistic sham conditions, which mimic the sensory stimulation of TMS but do not activate the cortex transcranially [82,83,84], or the removal of saliency-related vertex potentials via independent component analysis (ICA) [85,86]. 

**Figure 1:**
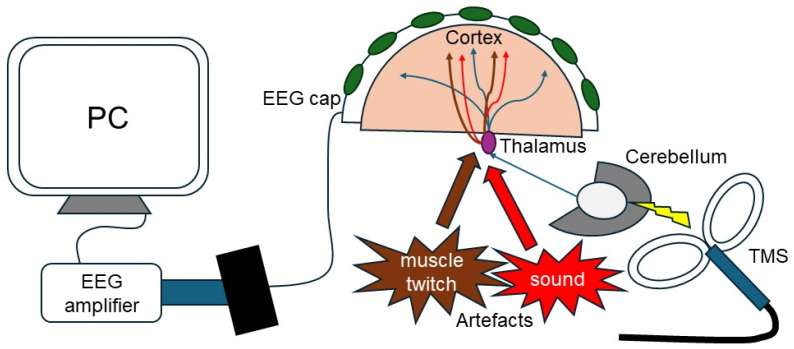
Concept and setup of cerebellar TMS-EEG recording and primary sources of sensory responses and artifacts.

The cerebellum has recently gained attention as a new target for TMS-EEG [86,87,88,89,90]. This interest arises from the limitations of functional MRI (fMRI) in providing sufficient temporal resolution to study rapid communication between hemispheres and the cerebellum [26], and of the classical CBI paradigm, which only assesses connections between cerebellum and contralateral M1. Cerebellar TMS-EEG (Figure 1) provides the potential to observe changes in the entire cortex with high temporal resolution and reasonable spatial resolution using high-density EEG, but it presents specific experimental challenges that differ from TMS-EEG over M1 [91,92]. This review aims to introduce available reports on cerebellar TMS evoked potentials (cbTEPs) and discuss current limitations and potential applications in this emerging field.

## 3. First Studies Investigating Cerebellar TMS Evoked Potentials

Early exploratory studies of cbTEPs were conducted at the single-channel level. In 1992, Amassian and colleagues observed early evoked potentials at 3.5 ms over one frontal channel (F3) and 8–13 ms over central regions (C3 and C4) following single-pulse cerebellar TMS. The stimulation was applied with a flat 50 mm figure-of-eight (F8) coil over the junction region between neck and head, at 45° away from the longitudinal axis of the neck [93]. Subsequent studies by Iramina and colleagues reported lateralized cbTEPs components with longer latencies, such as N10, P25, and N50, over F3 or F4 after contralateral single-pulse cerebellar TMS using a 70 mm F8 coil. Additionally, a P50 wave located at the vertex was found when TMS was applied 2 cm above the inion, but not dependent on the TMS [94,95,96]. Due to the small number of recording electrodes and the inconsistencies in latencies and polarity of cbTEPs, it is difficult to conclude the topography of cerebellar projections engaged by TMS from these early studies. 

More recent studies have used high-density EEG, with 64 or more electrodes, to record responses following cerebellar TMS in more detail. After stimulation of the I1 location (left of the inion) with a 70 mm F8 coil, one study by Arimatsu and coworkers [97] revealed a negative potential with 40–50 ms latency over the ipsilateral parietal region, with a corresponding positive wave over the contralateral parietal areas. A further report by Iwahashi and colleagues described positive deflections peaking around 20 ms in the frontal ipsilateral and contralateral parietal regions after cerebellar TMS targeted 3 cm left of the inion [98]. Notably, this study was the first to apply ICA for the rejection of TMS-induced artifacts. However, none of these reports addressed the issue of EEG responses due to sensory activation and they lacked sham stimulation conditions. In 2017, Du and coworkers compared cbTEPs obtained by stimulation, with a 70 mm F8 coil, placed over five different locations including the prefrontal cortex, vertex, left primary motor cortex, left primary auditory cortex, and cerebellum; the cerebellar target corresponded to the vermis and bilateral crus I/II. Although the spatial sampling was limited by the use of only 11 recording electrodes, the authors reported a frontal positive deflection at 18 ms, a frontal negative deflection at 25 ms, a frontal positive deflection from 40–80 ms, a central-frontal negative deflection from 110 to 130 ms, and a central positive deflection peaking around 190–220 ms [99]. Importantly, a sham coil auditory control condition was used in this experiment and produced vertex N100 and P200 peaks. 

## 4. Recent Research on Cerebellar TMS Evoked Potentials

More recent work on cbTEPs has applied more rigorous methodology, such as a systematic use of noise masking to suppress AEPs generated by the click of the coil [75,76]. Fernandez and coworkers showed that cerebellar activation induced by a 70 mm F8 coil is not sufficient to generate measurable EEG responses, even with high intensities (90% of the maximal stimulator output) [88]. However, the same group showed that a contralateral parietal negative deflection around 42 ms could be obtained by stimulation of the lateral cerebellum using a DC coil with a fixed stimulation intensity (60% MSO); this wave was not attributed to sensory activation, as it was not present in a sham control condition [88]. This observation aligns with previous literature indicating that cerebellar–brain inhibition (CBI) could not be produced by F8 coils [37,38,39].

Sasaki and colleagues [100] assessed EEG responses and CBI following single-pulse cerebellar stimulation with a DC coil and an F8 coil and added a “realistic” sham condition which included magnetic stimulation of the shoulder. As they were interested in how a stimulus to the cerebellum altered the TEP evoked by TMS over the motor cortex, they did not analyze the effect of cerebellar TMS alone. However, the figures in the paper appear to show a contralateral parietal positive deflection around 47 ms and a contralateral frontal positive deflection between 68 and 115 ms. No effective MEP inhibition was found when CBI was performed with an F8 coil over the cerebellum. Different from previous research, a later report by Gassmann and colleagues [89] employed a small, 50 mm flat F8 coil to stimulate the lateral cerebellum with a lower intensity, based on RMT from M1 (i.e., 75–76% MSO on average). A further difference with previous work is that cbTEPs were obtained by subtracting sham responses from real TMS, while applying supramaximal electrical stimulation to both, in an attempt to saturate somatosensory responses. CbTEPs in this work were represented by a contralateral frontal positive deflection around 25 ms and a contralateral parietal negative deflection around 45 ms, compared to the sham condition. Lastly, the cbTEPs obtained with stimulation of the lateral cerebellum by a DC coil in the study by Fong and coworkers consisted of contralateral frontal positive (80 ms, P80) and negative (110 ms, N110) deflections. These components were replicated by stimulating both cerebellar hemispheres and in different experimental sessions; importantly, a task-induced visuomotor adaptation correlated changes in the P80 and the degree of motor learning [86], thus providing evidence for a link between specific components of cbTEPs and cerebellar physiology. 

## 5. Cerebellar TMS Evoked Responses in the Frequency Domain

The literature on TMS-evoked responses assessed in the frequency domain is limited, but a few studies have explored this aspect. Schutter and colleagues [101] found increased theta power on a single EEG channel (AFz), between 200 and 4000 ms after a single TMS pulse over the vermis of the cerebellum, compared with sham and occipital stimulation. Du and colleagues [102] reported increased bilateral phase-locking value between F3 and F4 in the theta to gamma frequency range for 2 s after single-pulse cerebellar TMS over the midpoint between both cerebellar hemispheres, corresponding to crus I/II. This prefrontal synchrony was associated with GABA concentration detected by magnetic resonance spectroscopy (MRS) and correlated with performance in cognitive memory tasks. This study suggests that cerebellar TMS can influence functional connectivity in the prefrontal cortex across different frequency bands. The most recent work assessing cerebellar responses to TMS in the frequency domain is that of Gassmann and colleagues, who found increased left frontal theta power from 50–250 ms, decreased occipital alpha power from 250–550 ms, increased left hemispheric prefrontal power in the high-beta frequency band from 50 to 190 ms, and diffusely increased gamma power from 50–260 ms after single-pulse lateral cerebellar TMS, compared to sham stimulation, again using a sham method entailing saturation of somatosensory responses (see above) [89]. This study provides further insights into the frequency-specific changes induced by cerebellar TMS and highlights the complex dynamics of cortical responses in different frequency bands.

## 6. Discussion

### 6.1. Current Knowledge about EEG Responses to Cerebellar TMS

Table 1 lists the main findings mentioned in the previous paragraphs. Despite some inconsistencies observed across studies, some components of cbTEPs following TMS on cerebellar hemispheres have been at least partly reproduced, i.e., a P25, N45, and P80. A contralateral frontal P25 was identified in two studies using different methods [89,95], but was not confirmed elsewhere [86,88,100]. Similarly, a contralateral parietal N45 has been obtained in only two studies [88,89], but was not seen in other work [86,90,100], and in a single patient with damage to CTC connections [90]. A P80 has been consistently observed in experiments using DC coils at the intensity reaching the CBI effect [86,100], and its physiological relevance is supported by its relationship with visuomotor adaptation [86]. However, the absence of this component when stimulation is performed with low intensity [88] or F8 coils [88,89,100] suggests that it is highly dependent on experimental conditions.

In the frequency domain, an increase in neuronal oscillations over the frontal regions has been a consistent finding across three studies, albeit with variations in frequency bands and time windows, possibly due to differences in intensity and stimulating sites. Of particular note is the study by Du and colleagues [102], suggesting that cerebellar stimulation-induced bilateral prefrontal synchrony correlated not only with working memory and motor coordination performance, but also with Glutamine and GABA levels. Further research is essential to explore and elucidate the intricacies of cortical neuronal oscillations induced by cerebellar TMS.

The use of behavioral tasks involving the cerebellum and of patients with cerebellar lesions represents classical methods to understand the physiological and pathophysiological relevance of TMS markers of cerebellar function. This was originally done for CBI, when the cerebellar origin of the phenomenon was verified by applying it in patients with cerebellar stroke and ataxia [33,103,104,105]. Behavioral experiments have provided evidence to link long-term depression (LTD) of parallel fiber–Purkinje cell synapses induced by visuomotor adaptation [106] with CBI [40,41]. In addition, SEP components, such as the N18 or N24, have been used to probe the effect of motor learning on sensorimotor integration in the cortico-cerebellar network [107]. On similar grounds, Fong and coworkers found an increase in prefrontal P80 of cbTEPs after visuomotor adaptation, providing insights into the dynamics of cerebellar processing during motor learning through connections with cortical areas outside M1 [86]. New avenues for cbTEPs may be represented by behavioral tasks involving error processing; it has recently been reported that single-pulse cerebellar TMS influences the error-related negativity in go/no-go tasks [108]. Although this study does not primarily focus on cbTEPs, it underscores the broader potential of cerebellar TMS-EEG in neuroscience and behavior research. 

A fundamental question about cbTEPs revolves around their physiological meaning. Given that cbTEPs represent a cortical response obtained by stimulation of a remote structure, their nature likely differs from TEPs recorded at the stimulation site [51,53]. Conceptually, cbTEPs can be viewed as cortical post-synaptic potentials caused by the activity of CTC projections. The main output from the cerebellum is relayed by the dentate nucleus, which projects tonic facilitatory signals to the cortex via the ventral thalamus. In CBI, cerebellar stimulation activates cerebellar Purkinje cells, leading to inhibition of the dentate nucleus and subsequent, phasic suppression of cortical excitability [109]. Based on this assumption, cbTEPs components should at least in part reflect inhibitory post-synaptic potentials. Currently, there is no definitive answer to the nature of cbTEPs. Fong and coworkers suggested that the prefrontal P80 and, potentially, N110, might be a form of excitatory rebound phenomenon following stimulation of cerebellar Purkinje cells. This prolonged latency may also indicate that the P80 is not related to somatosensory input since cerebellar motor learning modulates much earlier components of the SEP [110]. Yet, more evidence is required to support and refine this hypothesis [86,92]

### 6.2. Discrepancies between EEG Responses to Cerebellar TMS across the Literature

We have highlighted above that there are significant discrepancies between the cbTEPs found by different research groups. Several factors may have contributed to this, the most likely being the different methods employed, such as TMS coil type, stimulation intensities, and solutions to suppress artifacts or sensory EEG responses.

The first crucial factor is the choice of TMS coil. The CBI protocol in which the cerebellum is stimulated with a DC coil is well established [33,103]. The DC coil is thought to activate Purkinje cells, leading to inhibition of tonic facilitatory output from the dentate nucleus of the cerebellum [109]. In contrast, cerebellar TMS with an F8 coil has mostly been considered ineffective in suppressing MEPs through CBI [36,37,38,100], although pulses applied with F8 coils over the cerebellum can still reduce M1 excitability by activating peripheral somatosensory inputs [109,111]. This difference between coils is supported by other observations. The depth of stimulation by the DC coil is deeper than that of the F8 coil, as confirmed by electric field measurements and modelling [86,112]. Thus, the evidence suggests that for single pulse cerebellar TMS, stimulation with the DC coil can activate the anterior cerebellum and contribute to the modulation of motor responses. In contrast, there is still no direct evidence showing where the F8 coil stimulates and whether it can produce neurophysiological responses in either anterior or posterior portions of the cerebellum. Fernandez and colleagues did not find significant TEPs when subtracting responses obtained from sham stimulation from those following cerebellar stimulation with a 70 mm F8 coil at 90% MSO [88]. Despite these findings, Gassmann and coworkers were able to elicit a contralateral P25 when stimulating the lateral cerebellum with an F8, 50 mm coil at 75% MSO. It is to be noted that a similar component was observed previously when TMS was applied 2 cm above the inion, a spot where the cerebellum is not preferentially activated [95,96]. As mentioned above, a contralateral P80 was observed only when the lateral cerebellum was stimulated with a DC coil with an intensity able to elicit CBI [86,100]. One reason why the same component was not observed by Fernandez and coworkers, despite the use of the same DC coil, might be due to the lower stimulation intensity (fixed at 60% MSO) in the absence of CBI assessment [88]. Another issue concerning stimulation is the targeting site, which varies among studies, including areas close to the neck [93], occipital areas [95,96], the cerebellar vermis [99,102], and lateral cerebellar sites optimal for CBI [86,88,89,97,98]. Overall, although the effects of coil type and stimulation site on cbTEPs still need to be systematically addressed, it is possible that by varying these factors, it is possible to activate different sets of cerebellar projections to cortex and thus to obtain cbTEPs with different spatial and temporal features. 

A further methodological aspect pertains to the simulation of the E-field generated by TMS, the importance of which was emphasised by Gassmann and coworkers [89]. However, the relationship between the E-field induced by TMS and induced neuronal firing is not simple, due to the interaction between the direction of the induced electric field and the neuronal orientation at the target site, and should not replace physiological measurements [26,92]. Modelling studies, as well as direct E-field measures, show that DC coils induce stronger and deeper E-fields compared to F8 coils [86,112]. Considering the greater effectiveness of DC coils compared to F8 coils in eliciting CBI [37,38,39], the results imply that CBI is more easily elicited with stronger and deeper E-fields such as those produced by DC coils.

The methods to eliminate or reduce artefacts and sensory responses caused by TMS present another potential issue in the mentioned studies. These confounds were not considered in research before 2009. As knowledge about how sensory input can contaminate TEPs emerged, the need to suppress the TMS click by using a masking noise and ear defenders has become clear [50,56,75,78]. However, it is challenging for subjects to wear ear defenders during cerebellar TMS, especially with DC coils [86]. At least two studies have shown that, in this experimental setting, completely suppressing the TMS click is not possible by only playing a masking noise through earphones, as indicated by the presence of a multimodal vertex N100/P200 [86,88]. Another possible confound is represented by somatosensory input, which is particularly large during cerebellar TMS due to local muscle contraction and, similar to the TMS click, may result in a multimodal vertex N100/P200 complex [81,86]. There is no routinely applicable method to suppress local muscle twitch and activation of free nerve endings during TMS [81]; therefore, most studies have devised sham conditions mimicking the somatosensory activation by TMS and compared them with real stimulation. However, the exact nature of stimulation varied, including electrical stimulation of muscles surrounding the stimulation site [86,88], combined magnetic stimulation of the shoulder and electrical stimulation of the neck [89], or the use of a sham coil [99]; the latter is imperfect, as common sham coils produce insufficient somatosensory input compared with active TMS coils [82,113]. An original solution to control for confounding factors was devised by Gassmann and colleagues, who applied supramaximal electrical stimulation in both real and sham conditions, with the objective of saturating SEPs, and then subtracted the second from the first to reveal the TEP [83,89]. However, this method may alter the spatiotemporal features of the evoked potential due to large unnecessary somatosensory inputs [83,92,114]. A different approach to dealing with possible EEG responses due to sensory input is their offline removal by ICA. This procedure has been applied in some reports to reduce vertex potentials evoked by auditory stimulation [70,85,86] and may theoretically be effective in reducing somatosensory responses as well, due to their spatial and temporal overlap [81]; however, further validation is needed before considering this method fully applicable. At present, there is still no optimal solution to control artifacts and sensory-evoked potentials in the context of cerebellar TMS. Therefore, an adequately designed control condition remains to be found. 

## 7. Conclusions

The study of cbTEPs is still a nascent field, and the development of new technologies for the suppression of artefacts and sensory responses remains a critical challenge for further research. While TMS-EEG measurements on the cortex have proven to be robust and reproducible, current findings related to cbTEPs are characterised by discrepancies in both time and frequency domains. Therefore, the establishment of consistent, optimised, user-friendly, and replicable protocols is essential for advancing both academic and clinical research in this area [91,92]. 

## 8. Perspectives

To gain more insights into cbTEPs, future studies might benefit from integrating behavioral assessments and patient-based models. Such multidisciplinary approaches hold the potential to unveil a deeper understanding of cbTEPs and pave the way for their broader application in neuroscience and clinical research.

## Figures and Tables

**Table 1:** Summary of findings in studies assessing cbTEPs. F8: 70 mm figure-of-eight coil. DC: 110 mm double cone coil. sF8: 50 mm figure-of-eight coil. Asterisks indicate that findings were qualitatively assessed but not further analyzed. * indicates that these is only observations, not statistical results.

Study	Findings	Coil
Single channel Studies		
Amassian, 1992 [93]	3.5 ms at F3, 8–13 ms at C3/C4	sF8
Iramina, 2002 [94]	P50 on Cz	F8
Iramina, 2003 [95], 2004 [96]	N10, P25, N50 at F3/F4	F8
Du, 2017 [99]	Frontal P18, N25, P40–80, N100, central P200	F8
High-density EEG studies		
Arimatsu, 2007 [97]	Ipsilateral parietal N20, contralateral parietal P40	F8
Iwahashi, 2009 [98]	Ipsilateral frontal and contralateral parietal P20	F8
Fernandez, 2021 [88]	Contralateral parietal N42	DC
	No significant findings	F8
Sasaki, 2022 [100]	Contralateral positive deflection 68–105 ms *	DC
	Contralateral positive parietal deflection at 47 ms *	F8
Gassmann, 2022 [89]	Contralateral frontal P25 and parietal N45	sF8
Fong, 2023 [86]	Contralateral frontal P80-N110, correlated with motor learning	DC
